# Regional Brain Stem Atrophy in Idiopathic Parkinson's Disease Detected by Anatomical MRI

**DOI:** 10.1371/journal.pone.0008247

**Published:** 2009-12-10

**Authors:** Thomas Jubault, Simona M. Brambati, Clotilde Degroot, Benoît Kullmann, Antonio P. Strafella, Anne-Louise Lafontaine, Sylvain Chouinard, Oury Monchi

**Affiliations:** 1 Unité de Neuroimagerie Fonctionelle, Institut Universitaire de Gériatrie de Montréal, Montreal, Quebec, Canada; 2 Département de Radiologie, Université de Montreal, Montreal, Quebec, Canada; 3 Toronto Western Hospital/Research Institute & CAMH-PET Imaging Centre, University of Toronto, Toronto, Ontario, Canada; 4 Movement Disorders Unit, McGill University Health Center, Montreal, Quebec, Canada; 5 Unité des Désordres du Mouvement, Centre hospitalier de l'Université de Montréal, Montreal, Quebec, Canada; University of Nebraska, United States of America

## Abstract

Idiopathic Parkinson's disease (PD) is a neurodegenerative disorder characterized by the dysfunction of dopaminergic dependent cortico-basal ganglia loops and diagnosed on the basis of motor symptoms (tremors and/or rigidity and bradykinesia). Post-mortem studies tend to show that the destruction of dopaminergic neurons in the substantia nigra constitutes an intermediate step in a broader neurodegenerative process rather than a unique feature of Parkinson's disease, as a consistent pattern of progression would exist, originating from the medulla oblongata/pontine tegmentum. To date, neuroimaging techniques have been unable to characterize the pre-symptomatic stages of PD. However, if such a regular neurodegenerative pattern were to exist, consistent damages would be found in the brain stem, even at early stages of the disease. We recruited 23 PD patients at Hoenn and Yahr stages I to II of the disease and 18 healthy controls (HC) matched for age. T1-weighted anatomical scans were acquired (MPRAGE, 1 mm3 resolution) and analyzed using an optimized VBM protocol to detect white and grey matter volume reduction without spatial a priori. When the HC group was compared to the PD group, a single cluster exhibited statistical difference (p<0.05 corrected for false detection rate, 4287 mm3) in the brain stem, between the pons and the medulla oblongata. The present study provides in-vivo evidence that brain stem damage may be the first identifiable stage of PD neuropathology, and that the identification of this consistent damage along with other factors could help with earlier diagnosis in the future. This damage could also explain some non-motor symptoms in PD that often precede diagnosis, such as autonomic dysfunction and sleep disorders.

## Introduction

Idiopathic Parkinson's disease (PD) is a neurodegenerative disorder characterized by the progressive loss of dopaminergic neurons of the subtantia nigra pars compacta, and clinically diagnosed on the basis of a motor symptomatology: tremor, rigidity and/or bradykynesia.

PD clinical diagnosis is particularly prone to errors [1], [2], as this array of motor symptoms is also present in a wide range of other parkinsonian conditions such as progressive supranuclear palsy, dementia with Lewy bodies, and multiple system atrophy. Confirmation of the diagnosis is often brought by disease progression and the response of patients to levodopa medication.

To date, in-vivo methods of brain imaging, CT-scan and magnetic resonance imaging (MRI), have demonstrated little to no sensitivity to PD. Recent analysis methods based on structural MRI (T1-weighted contrast, diffusion tensor imaging [3]) have been able to exhibit significant differences between demented and non-demented PD patients [4], [5], or between PD and PSP [6] or dementia with Lewy bodies patients [7]. While these studies brought some insight to the differential diagnosis of these various diseases, they have been unable so far to clearly distinguish PD patients from healthy controls [8], [9].

Furthermore, post-mortem studies by Braak and colleagues [10], [11], based on the analysis of Lewy bodies and neurites accumulation, a proteic hallmark of PD, have shown that various cerebral structures are damaged before subtantia nigra in a consistent and repeated pattern. They suggest that the diagnosis temporal window of PD comes up to 10 years after the first structural damages caused by the disease. In a 6 stages model [12], PD would initially begin in the medulla oblongata and in the olfactory bulb and progress in a caudorostral pattern, affecting subtantia nigra in stage 3 only, corresponding to the onset of the motor symptoms and the first visit of the patient to a neurologist. Degeneration of pontine tegmentum nuclei antecedent to the death of nigral dopaminergic neurons would generate a prior array of non-motor symptoms, such as rapid eye movement sleep behavior disorder (RBD), a condition that causes the loss of atonia during sleep. RBD is incidentally found in more than half PD patients [13].

The aim of the present study is to characterize morphological differences between PD patients at an early stage of the disease and healthy controls using standard MRI scans and procedures. In the framework of Braak's progression of the disease, we should observe damages across all PD patients localized in the brainstem, the hypothesized initial location of the neurodegeneration. Voxel based morphometry (VBM) is a technique that identifies local atrophies without the subjective selection of an a priori region of interest. Based on previous neuropathological findings, we expected to observe brain tissue volume reduction in the medulla oblongata/lower pontine tegmentum of PD patients compared to healthy control subjects.

## Methods

All participants gave informed consent to the protocol, which was reviewed and approved by the Joint Ethics Committee of the Regroupement Neuroimagerie Québec (RNQ). Twenty-three patients diagnosed with Parkinson's disease participated in the study, as well as 18 healthy controls matched for age. All PD participants met the core assessment program for surgical interventional therapy criteria for the diagnosis of idiopathic PD [14], [15] and the UK brain bank criteria for the diagnosis of Parkinson's disease [16]. Motor disability of individuals within the PD group was in the mild to moderate severity range according to the Hoehn and Yahr staging criteria [17]. The Montreal Cognitive Assesment [18] was used to screen for early signs of dementia.

### MRI Scanning

Participants were scanned using a 3T Siemens Trio MRI scanner at the Functional Neuroimaging Unit, at the Research Center of the Montreal Geriatric Institute (TR/TE/TI: 2300/2.91/900 ms, Flip angle: 9°, 160 slices, field of view: 256×240 mm, matrix: 256×240, voxel size: 1×1×1 mm, 12-channels coil).

VBM analysis included two steps: spatial preprocessing (normalization, segmentation, Jacobian modulation and smoothing) and statistical analysis. Both steps were implemented in the SPM software package [19] (Wellcome Department of Imaging Neuroscience, London; http://www.fil.ion.ucl.ac.uk/spm) running on Matlab 7.3 (MathWorks, Natick, MA).

MRI images were pre-processed using an optimized standard procedure [20]. A study-specific template and a priori images were created by averaging all the anatomical scans that had been normalized and segmented in the MNI (Montreal Neurological Institute) stereotaxic space. A two-step segmentation procedure was then applied to the scans in this analysis. First, T1-weighted images were segmented in native space. Each grey/white matter image was then normalized to the grey/white matter template. The parameters obtained from the white-matter normalization were then applied to the original T1 images. Finally, the normalized images were segmented again into grey matter, white matter and cerebrospinal fluid. White and grey matter voxel values were multiplied by the Jacobian determinants derived from the spatial normalization step (Jacobian modulation) to preserve the initial volumes, to obtain modulated white matter images. These images were then spatially smoothed with a 12 mm FWHM isotropic Gaussian kernel. This kernel size was shown to minimize the risk of false positive findings [21].

The normalized, segmented, and smoothed data were statistically tested using a general linear model based on gaussian field theory using analysis of covariance, with age and gender of participants and total amount of white matter (WM) or grey matter (GM) volume treated as nuisance covariates to detect regional areas of relative accelerated loss of WM or GM volume. The statistical threshold was set at P<0.05, using false discovery rate correction for multiple comparisons at the voxel level, and so was the statistical threshold at the cluster level.

We applied the exact same methods to grey matter maps to verify the absence of significant volume variation between the two groups similarly to what has been previously reported in the literature.

## Results

A total of 23 PD patients (mean age = 64.02±5.47 years, 10 females, mean duration since first diagnosis = 6.33±3.93 years) and 18 healthy controls (mean age = 62.17±5.40 years, no significant differences with the PD group, 10 females) were evaluated. The motor subset of the Unified Parkinson Disease Rating Scale III was assessed to measure motor symptoms in the PD group after a 12-hours withdrawal of anti parkinsonian medication ( mean score = 29.07±8.97).

**Figure 1:**
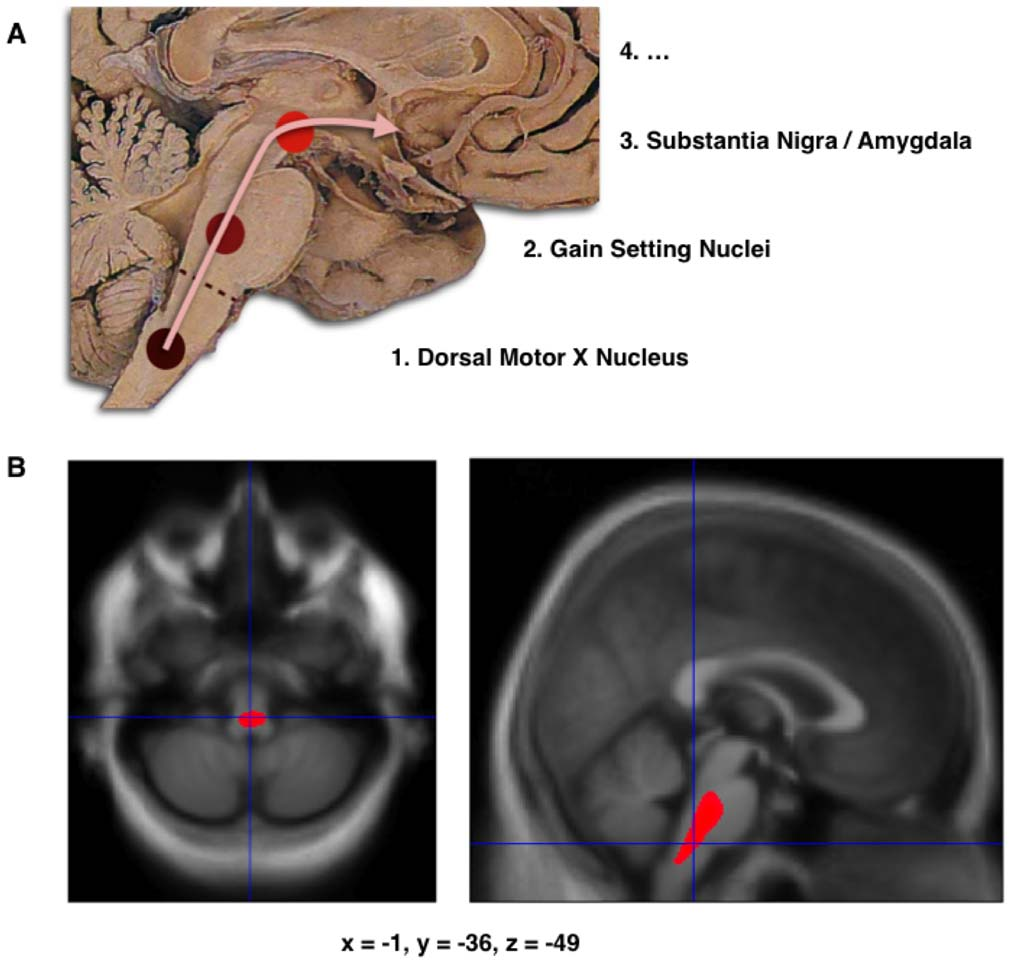
Localization of the atrophy. A. Schematic initial progression of Lewy body deposits in the first stages of Parkinson's Disease, as proposed by Braak and colleagues. B. Localization of the cluster of significant volume reduction in PD compared with HC. The significant cluster located in the medulla oblongata/pons is superimposed as a red blob on the mean normalized anatomical scan of all participants. The axial and sagital sections are centered on the peak of significance (−1; −36; −49).

**Figure 2:**
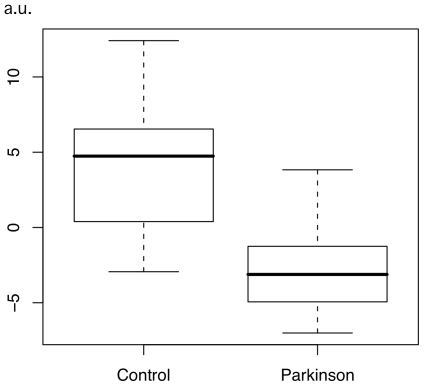
Effect size at the peak of significance. Boxplot of the peak of significance value (arbitrary unit) for each participant in both groups, as obtained by SPM5 ‘fitted response’ plot function. Peak value was adjusted, meaning that the effect of age, gender and global WM volume were factored out, and only the effect of condition (Parkinson or Control) and the residuals remained, as assessed by the SPM model. For each group, bold line shows the median, central box includes the middle 50% of the data, and tails of the boxplot show the minimum and maximum values.

When comparing the modulated WM maps of the healthy control with the PD group, a single symmetrical cluster was found, in the caudal part of the pons, overlapping with the rostral medulla oblongata (figure 1 and 2, volume = 4287 mm3, peak coordinate in the MNI space: -1, -36, −49, p<0.05 corrected for multiple comparisons by false discovery rate, T score = 6.40, Z score = 5.17, cluster significant at p<0.05 corrected for non-stationarity, note that the peak survives a p<0.05 family-wise error statistical threshold). No other cluster was found at that significance level, or in the converse contrast.

We ran additional analyses to assess the effect of age in the tested population. No cluster of significant atrophy survived at the threshold used. At last, we ran analyses specific to the PD groups, using age, gender, UPDRS-III score and duration of the disease (expressed as the time elapsed between the first neurological diagnosis and the MRI acquisition) as covariates, and found no atrophy patterns. However, it should be noted that in the PD group, at a low threshold of 0.001 uncorrected for multiple comparisons, and no correction for cluster size, a symmetrical cluster of age-related atrophy was observed in the middle corpus callosum.

Finally, in the GM maps, no significant differences were found between the two groups even at a low threshold of 0.001 uncorrected for multiple comparisons.

## Discussion

The goal of the study was to characterize the volume reduction pattern in white matter associated with PD.

The key result is the finding of a single significant cluster of white matter volume reduction, overlapping the rostral part of the medulla oblongata and the caudal part of the pons. This finding is consistent with the existence of a common anatomical starting point of PD, in accordance with Braak et al. [10], [12], unrelated to the duration of the disease, or the severity in term of motor impairments. This finding is also independent of age, although this factor is known to interact with PD condition [22]. In the present study, sub-threshold, age-related WM atrophy was noticed in the PD group, but not in the brainstem.

White matter hyperintensities that have been observed previously in anatomical MRI volumes of PD patients [23] are known to cause segmentation errors. However, these hyperintensities are bound to periventricular territories, and could not have affected the findings of the present study.

Registration errors occurring during the normalization of individual scans is a common criticism of the VBM method [24], as voxels close on a spatial scale, but distant in term of cerebral architecture (for instance, two different white matter bundles spreading in one part and another of a sulcus) could be merged during the analysis. However, the tubular geometric nature of the brainstem preserves our result from this drawback.

It is of particular interest that we found no other white or grey matter region than the lower brain stem exhibiting signs of atrophy at the group level. Since VBM analysis is a conservative method, as no a priori region of interest is studied, we postulate that damage in this region is common to all patients while the regional evolution of the neurodegenerative process may be more variable across PD patients, and hence lowers the sensibility of white matter VBM analysis in other brain regions. It is also likely that later stages of the disease affect grey matter more significantly but in a more heterogeneous way. Indeed previous studies using VBM in PD patients concentrated solely on grey matter and could not find any significant differences with healthy individuals [8]. Our results on grey matter also confirm these findings.

It should be noted that the automatic segmentation algorithm we used classifies brainstem as white matter, in spite of grey matter presence. The result we observe should therefore be considered with care, as an array of different microanatomical factors could be at the origin of this decrease. Lewy bodies have been historically considered as the hallmark of the disease [25], and more recently, their presence in the substantia nigra has been proposed to correlate with the motor scores of the UPDRS [26]. However, recent progresses in molecular biology and histo-immunology suggest that alpha-synuclein deposits, which constitute the main part of Lewy bodies, involve other cerebral structures than those latter, including the pons and the medulla oblongata, and that it should be considered as a more direct measure of PD pathology and clinical symptomatology [27]. Finally, while we can not conclude regarding the white or grey matter fine origin of the VBM decrease observed in this study, it should also be noted that alpha-synuclein deposits has been shown to affect white matter in the Multi-Systemic Atrophy [28].

In conclusion, to our knowledge, this is the first study demonstrating that conventional T1-weighted 3 T MRI scans can validate in vivo, on a group basis, the first stages of Braak's model [10]–[12], This key finding, based on conventional MRI scans easily acquired in clinical practice, could define a critical marker of the disease, in association with other biomarkers, for the diagnosis of PD when initial symptomatology appears, but also presymptomatically as other related symptoms occurs, such as autonomic dysfunctions[29], RBD [13], or anosmia [30]. Future studies should investigate the existence of a brain stem volumetric reduction in related diseases, such as Lewy body dementia or other forms of Parkinsonism. In order to validate the method further, these studies should also be combined with methods that measure the degree of dopamine denervation in those patients such as 11C-DTBZ PET in North America [31] or DATscan in Europe. They should also use larger population samples in association with markers extracted from other MRI modalities (such as diffusion tensor imaging, magnetization transfer ratio), in order to test the diagnostic validity of the present finding in PD.

## References

[1] Tolosa E, Wenning G, Poewe W (2006). The diagnosis of Parkinson's disease.. Lancet neurology.

[2] Meara J, Bhowmick BK, Hobson P (1999). Accuracy of diagnosis in patients with presumed Parkinson's disease.. Age and ageing.

[3] Blain CR, Barker GJ, Jarosz JM, Coyle NA, Landau S (2006). Measuring brain stem and cerebellar damage in parkinsonian syndromes using diffusion tensor MRI.. Neurology.

[4] Ramírez-Ruiz B, Martí MJ, Tolosa E, Bartrés-Faz D, Summerfield C (2005). Longitudinal evaluation of cerebral morphological changes in Parkinson's disease with and without dementia.. J Neurol.

[5] Summerfield C, Junqué C, Tolosa E, Salgado-Pineda P, Gómez-Ansón B (2005). Structural brain changes in Parkinson disease with dementia: a voxel-based morphometry study.. Archives of Neurology.

[6] Price S, Paviour D, Scahill R, Stevens J, Rossor M (2004). Voxel-based morphometry detects patterns of atrophy that help differentiate progressive supranuclear palsy and Parkinson's disease.. Neuroimage.

[7] Burton EJ, McKeith IG, Burn DJ, Williams ED, O'Brien JT (2004). Cerebral atrophy in Parkinson's disease with and without dementia: a comparison with Alzheimer's disease, dementia with Lewy bodies and controls.. Brain.

[8] Seppi K, Schocke MFH (2005). An update on conventional and advanced magnetic resonance imaging techniques in the differential diagnosis of neurodegenerative parkinsonism.. Curr Opin Neurol.

[9] Nagano-Saito A, Washimi Y, Arahata Y, Kachi T, Lerch JP (2005). Cerebral atrophy and its relation to cognitive impairment in Parkinson disease.. Neurology.

[10] Braak H, Del Tredici K, Rüb U, De Vos RA, Jansen Steur EN (2003). Staging of brain pathology related to sporadic Parkinson's disease.. Neurobiology of Aging.

[11] Del Tredici K, Rüb U, De Vos RA, Bohl JR, Braak H (2002). Where does parkinson disease pathology begin in the brain?. J Neuropathol Exp Neurol.

[12] Braak H, Ghebremedhin E, Rub U, Bratzke H, Del Tredici K (2004). Stages in the development of Parkinson's disease-related pathology.. Cell Tissue Res.

[13] Gagnon JF, Bédard MA, Fantini ML, Petit D, Panisset M (2002). REM sleep behavior disorder and REM sleep without atonia in Parkinson's disease.. Neurology.

[14] Langston JW, Widner H, Goetz CG, Brooks D, Fahn S (1992). Core assessment program for intracerebral transplantations (CAPIT).. Mov Disord.

[15] Defer GL, Widner H, Marié RM, Rémy P, Levivier M (1999). Core assessment program for surgical interventional therapies in Parkinson's disease (CAPSIT-PD).. Mov Disord.

[16] Hughes AJ, Daniel SE, Kilford L, Lees AJ (1992). Accuracy of clinical diagnosis of idiopathic Parkinson's disease: a clinico-pathological study of 100 cases.. J Neurol Neurosurg Psychiatry.

[17] Hoehn MM, Yahr MD (1967). Parkinsonism: onset, progression and mortality.. Neurology.

[18] Nasreddine ZS, Phillips NA, Bédirian V, Charbonneau S, Whitehead V (2005). The Montreal Cognitive Assessment, MoCA: a brief screening tool for mild cognitive impairment.. Journal of the American Geriatrics Society.

[19] Friston KJ, Holmes AP, Worsley KJ, Poline JB (1995). Statistical parametric maps in functional imaging: a general linear approach.. Hum Brain Mapp.

[20] Good CD, Johnsrude IS, Ashburner J, Henson RNA (2001). A Voxel-Based Morphometric Study of Ageing in 465 Normal Adult Human Brains.. Neuroimage.

[21] Salmond CH, Ashburner J, Vargha-Khadem F, Connelly A, Gadian DG (2002). Distributional assumptions in voxel-based morphometry.. Neuroimage.

[22] Levy G (2007). The relationship of Parkinson disease with aging.. Arch Neurol.

[23] Sohn YH, Kim JS (1998). The influence of white matter hyperintensities on the clinical features of Parkinson's disease.. Yonsei Med J.

[24] Bookstein F (2001). “Voxel-Based Morphometry” Should Not Be Used with Imperfectly Registered Images.. NeuroImage.

[25] Escourolle R, de Recondo J, Grey F (1970). Aspects neuropathologiques des syndromes parkinsoniens.. Rev Prat.

[26] Greffard S, Verny M, Bonnet AM, Beinis JY, Gallinari C (2006). Motor score of the Unified Parkinson Disease Rating Scale as a good predictor of Lewy body-associated neuronal loss in the substantia nigra.. Arch Neurol.

[27] Duyckaerts C, Verny M, Hauw JJ (2003). [Recent neuropathology of parkinsonian syndromes].. Rev Neurol (Paris).

[28] Ghorayeb I, Bezard E, Fernagut PO, Bioulac B, Tison F (2005). [Animal models of parkinsonism].. Rev Neurol (Paris).

[29] Chaudhuri KR (2001). Autonomic dysfunction in movement disorders.. Curr Opin Neurol.

[30] Ward CD, Hess WA, Calne DB (1983). Olfactory impairment in Parkinson's disease.. Neurology.

[31] Kumar A, Mann S, Sossi V, Ruth TJ, Stoessl AJ (2003). [11C]DTBZ-PET correlates of levodopa responses in asymmetric Parkinson's disease.. Brain.

